# Maintained improvement in patient-reported outcomes in early axial spondyloarthritis following certolizumab pegol dose reduction: findings from the randomised period of a phase 3b trial

**DOI:** 10.1016/j.ero.2026.100183

**Published:** 2026-05-07

**Authors:** Antoni Chan, Jon Wood, Lachrissa Burns, Thomas Kumke, Bengt Hoepken, Mindy Kim, Lars Bauer, Kirsten Mackay, Karl Gaffney

**Affiliations:** 1 University Department of Rheumatology, Royal Berkshire NHS Foundation Trust, Reading, UK; 2 Immunology Division, UCB Pharma, Slough, UK; 3 Immunology and Biosciences Division, UCB BioSciences GmbH, Monheim/Rhein, Germany; 4 Immunology Division, UCB, Atlanta, Georgia, USA; 5 Department of Rheumatology, Torbay and South Devon NHS Foundation Trust, Torquay, UK; 6 Department of Rheumatology, Norfolk and Norwich University Hospitals, NHS Foundation Trust, Norwich, UK

## Abstract

**Background:**

In the phase 3b C-OPTIMISE study (ClinicalTrials.gov, NCT02505542), approximately 80% of patients with early active axial spondyloarthritis (axSpA) remained flare-free from week (W)48 to W96 with full-dose and reduced-dose certolizumab pegol (CZP) after gaining sustained remission (Axial Spondyloarthritis Disease Activity Score <1.3 at W32/36, and W48; if <1.3 at W32, <2.1 was required at W36, or vice versa). Patient-reported outcomes (PROs) are presented throughout the maintenance period for patients in sustained remission at W48.

**Methods:**

After open-label CZP through W48, patients were double-blind randomised 1:1:1 to full-dose CZP (200 mg every 2 weeks [Q2W]), reduced-dose CZP (Q4W), or placebo. Prespecified PROs: Bath Ankylosing Spondylitis Disease Activity Index (BASDAI), Bath Ankylosing Spondylitis Functional Index (BASFI). *Post hoc*: Patient’s Global Assessment of Disease Activity (PtGADA), nocturnal spinal pain (NSP), and Ankylosing Spondylitis Quality of Life (ASQoL).

**Results:**

At W48, 43.9% of patients (323/736) achieved sustained remission. At timepoints from W48 through W96, PRO scores were maintained with full-dose and reduced-dose CZP, vs rapid increases (worsening) with placebo. At W96, least squares mean score change from W48 with full-dose and reduced-dose CZP vs placebo (*P* < .001) was: BASDAI total 0.56 and 0.78 vs 3.02; BASDAI morning stiffness 0.58 and 0.73 vs 3.56; BASDAI fatigue 0.52 and 0.70 vs 2.12; BASFI 0.32 and 0.46 vs 1.90; PtGADA 0.67 and 0.98 vs 3.65; NSP 0.62 and 0.80 vs 2.60; and ASQoL 0.54 and 0.86 vs 4.28.

**Conclusions:**

In patients who achieved remission at W48, reduced-dose and full-dose CZP maintained axSpA in remission without impacting PROs through W96.

**WHAT IS ALREADY KNOWN ON THIS TOPIC**

• Tumour necrosis factor inhibitors (TNFi) are used to induce and maintain remission in patients with axial spondyloarthritis (axSpA).
• International guidelines recommend considering dose reduction of biologic therapy for patients in sustained remission. These guidelines also recommend that disease monitoring should include patient-reported outcomes (PROs), in addition to clinical findings, laboratory investigations, and imaging.
• The C-OPTIMISE phase 3b trial of certolizumab pegol (CZP), a TNFi, comprised 2 consecutive 48-week periods: an open-label induction period (baseline to week 48) and a randomised, double-blind, maintenance period (week 48-96).
• The study demonstrated that, after gaining sustained remission at week 48, reduced-dose CZP can maintain axSpA remission in a comparable way to full-dose CZP from week 48 to 96.

**WHAT THIS STUDY ADDS**

• Here, we report PRO findings from the randomised maintenance period of C-OPTIMISE, in patients with axSpA in remission at week 48, demonstrating that the previously reported maintained improvements in disease control from week 48 to 96 with reduced-dose CZP can be achieved without compromising PRO scores.

**HOW THIS STUDY MIGHT AFFECT RESEARCH, PRACTICE OR POLICY**

• These findings offer reassurance to patients and physicians that overall quality of life should, in general, not be impacted by reducing the CZP dose in those patients who have achieved sustained remission.

Alt-text: Unlabelled box dummy alt text

## INTRODUCTION

Axial spondyloarthritis (axSpA) is a chronic inflammatory rheumatic disease of the spine and sacroiliac joints [1]. Typically manifesting in the third decade of life, axSpA is categorised into radiographic axSpA (ie, with radiographic sacroiliitis; also termed ankylosing spondylitis) and nonradiographic axSpA (without evidence of radiographic sacroiliitis) [1]. Common symptoms of both axSpA subtypes include spinal pain, stiffness, and fatigue [[1], [2], [3]]. These manifestations can substantially affect a patient’s health, with impacts on physical function, mental health, social interaction, work productivity, and overall quality of life (QoL) [2,3]. The burden of disease is comparable for radiographic and nonradiographic axSpA [3,4].

A key treatment option for many patients with axSpA is tumour necrosis factor inhibitor (TNFi) therapy [5]. TNFi can induce and maintain remission (or low disease activity), which is typically accompanied by improvements in QoL [6,7]. International guidelines, updated in 2022 and 2025, recommend considering biologic tapering in patients with axSpA who demonstrate sustained remission, which may be based on inactive (<1.3) or low disease activity (<2.1) on the Axial Spondyloarthritis Disease Activity Score (ASDAS) scale [8,9]. The option to reduce TNFi dose is important, as long-term TNFi treatment is associated with considerable costs [10] and the possibility of adverse events and long-term immunosuppression [11]. Consequently, TNFi treatment strategies are under scrutiny with respect to how best to maintain remission in patients with autoimmune diseases, including axSpA.

Certolizumab pegol (CZP), a PEGylated TNFi without the Fc region, has demonstrated favourable long-term efficacy and safety in patients with axSpA [6,12]. Until recently, there were no formal studies of TNFi dose-reduction strategies in a broad axSpA population or within the radiographic/nonradiographic subtypes. C-OPTIMISE, a 2-part, phase 3b trial, investigated CZP dose reduction in this broad axSpA population [[13], [14], [15]]. The study demonstrated that, at week 48 of the induction period, 43.9% of patients achieved sustained remission (based on ASDAS scores) with open-label CZP therapy [13,14]. Moreover, in patients who achieved sustained remission by week 48 with open-label CZP therapy, the CZP dose could be successfully reduced without compromising disease control during the double-blind randomised maintenance period (week 48-96) [14]. In particular, based on investigator and patient assessments of ASDAS, the proportion of patients in the overall population who remained flare-free throughout the maintenance period (primary endpoint) was significantly higher with both full-dose CZP (83.7%) and reduced-dose CZP (79.0%), compared with CZP withdrawal (20.2%; *P* < .001) [14].

Evaluation of patient-reported outcomes (PROs) is an important aspect of dose reduction, as patients may be concerned that their QoL will worsen with reduced-dose therapy. Moreover, international guidelines recommend that disease monitoring should include PROs, in addition to clinical findings, laboratory investigations, and imaging [8] The C-OPTIMISE study demonstrated substantial improvements in PROs with CZP therapy during the induction period (from baseline to week 48), including in the Bath Ankylosing Spondylitis Disease Activity Index (BASDAI), nocturnal spinal pain, and Ankylosing Spondylitis Quality of Life (ASQoL) scales [13,15]. These PRO scales are used to assess various symptoms and physical functioning (eg, fatigue, spinal and joint pain, and morning stiffness) and the impact of axSpA on QoL. Here, changes in various PRO scores were assessed at timepoints throughout the maintenance period of C-OPTIMISE (week 48-96) to determine whether CZP dose reduction impacted PROs following achievement of remission at week 48.

## METHODS

### Study design

C-OPTIMISE was a phase 3b, 2-part, multicentre study evaluating axSpA remission and PROs during treatment with CZP (ClinicalTrials.gov, NCT02505542; registered in July 2015). The study comprised 2 consecutive 48-week periods: an open-label induction period (baseline to week 48) and a randomised, parallel-group, double-blind, placebo-controlled maintenance period (week 48-96). Details of the study design have been published previously [13,14].

Patients were screened at 108 study sites in the United States, Europe, and Asia (Supplementary Table S1). The study was approved by institutional review boards and independent ethics committees for each study site (listed in a previous publication) [13]. It was conducted in accordance with local regulations, the International Conference on Harmonisation, Good Clinical Practice, and the Declaration of Helsinki. All patients provided written informed consent to participate.

### Patients

Full inclusion and exclusion criteria have been published [13,14]. In brief, patients were adults (18-45 years of age) with early active axSpA (radiographic or nonradiographic) with symptom duration ≥3 months and <5 years and meeting the Assessment of SpondyloArthritis International Society (ASAS) classification criteria [16]. Active disease was defined as ASDAS ≥2.1, BASDAI ≥4, and spinal pain ≥4 on a 0 to 10 numerical rating scale (NRS), ie, BASDAI item 2.

### Study procedures and definitions

During the 48-week open-label induction period, all patients received subcutaneous CZP 200 mg every 2 weeks (Q2W), after a loading dose of 400 mg at weeks 0, 2, and 4. Patients who achieved sustained remission at week 48 were eligible to enter the subsequent 48-week double-blind maintenance period. Sustained remission was defined as ASDAS [17,18] <1.3 (inactive disease) either at week 32 or 36, and at week 48; if the score was <1.3 at week 32, a score of <2.1 (low disease activity) was required at week 36, or vice versa.

At baseline (week 48) in the double-blind maintenance period, patients in sustained remission were randomised (1:1:1) to continue on full-dose CZP (200 mg Q2W) or receive reduced-dose CZP (200 mg every 4 weeks [Q4W]) or placebo, with final doses administered at week 94. Randomisation was stratified by geographical region (Supplementary Table S1) and the presence or absence of radiographic sacroiliitis. Additional information on randomisation, study treatment, and blinding is shown in Supplementary Table S2.

The maintenance period included an early escape arm, in which patients experiencing a flare received open-label CZP 200 mg Q2W (patients from the placebo group initially received CZP 400 mg Q2W at 3 consecutive visits in the escape arm). Flare was defined as ASDAS ≥2.1 (high disease activity) at 2 consecutive visits or ASDAS >3.5 (very high disease activity) at any visit. Results for the escape arm have been published [14].

### Patient and public involvement

Patients were not involved in the study design or conduct. A lay summary reporting published study outcomes is available on the study sponsor website.

### Outcome measures and endpoints

The PRO scales included in the current analyses of the 48-week maintenance period were: BASDAI total score (calculated from 6 BASDAI subscale scores) [19], BASDAI subscales for morning stiffness and spinal pain [19], Bath Ankylosing Spondylitis Functional Index (BASFI) [20], Patient’s Global Assessment of Disease Activity (PtGADA) [21], nocturnal spinal pain score [21], and ASQoL [22].

The prespecified PRO endpoints reported herein (ie, change from randomisation baseline [week 48] in BASDAI and BASFI) were added to the study in a protocol amendment in November 2015, although BASDAI and BASFI endpoints in patients experiencing flares during the maintenance period (who entered the escape arm) were included in the original version of the protocol and were reported previously [14]. Other PRO endpoints (ie, change from randomisation baseline in PtGADA, nocturnal spinal pain score, and ASQoL) were analysed *post hoc*. These PRO analyses were added to the study to comprehensively investigate the effects of CZP therapy from patients’ perspectives.

Higher BASDAI total and subscale scores (ranging from 0-10) reflect worse disease activity. BASDAI total scores are based on 6 NRS, with questions to assess the severity of fatigue (Q1), spinal pain (Q2), peripheral joint pain (Q3), localised tenderness (Q4), and the severity and duration of morning stiffness (Q5 and Q6). To calculate BASDAI total scores, the sum of Q5 and Q6 scores was divided by 2 and added to scores from Q1 to 4, which was divided by 5. Higher BASFI score (ranging from 0-10) reflects worse physical functioning, based on the 10 NRS. Higher PtGADA score (ranging from 0-10) reflects worse disease activity. A higher nocturnal spinal pain NRS score (ranging from 0-10) reflects worse pain. ASQoL is an 18-item questionnaire, with a higher score (ranging from 0-18) reflecting greater impact on QoL.

In addition to PROs, other outcomes that were assessed across the maintenance period included treatment compliance (number of administered syringes, compared with the scheduled number of injections) and the percentages of patients treated with concomitant nonsteroidal anti-inflammatory drugs (NSAIDs) and disease-modifying antirheumatic drugs (DMARDs). ASAS-NSAID intake scores were also calculated *post hoc*. Higher ASAS-NSAID scores (ranging from 0-100) indicate greater intake of NSAIDs (the type of NSAID, dose, and number of days of intake were included in the calculation).

Primary and secondary efficacy and safety outcomes have been reported previously [13,14]. The primary endpoint was the percentage of patients remaining flare-free during the maintenance period, which was statistically tested to establish superiority for CZP over placebo.

### Schedule of assessments

In the current analyses, changes in PRO scores from week 48 were evaluated for BASDAI and PtGADA (at weeks 50, 52, 56, 60, 64, 68, 72, 76, 80, 84, 88, 92, and 96), nocturnal spinal pain (at weeks 52, 60, 72, 84, and 96), and BASFI and ASQoL (at weeks 60, 72, 84, and 96).

### Statistical analysis

In the maintenance period, changes in PRO scores were assessed using observed cases and mixed models for repeated measures (MMRMs). Each MMRM analysis included all observed postbaseline data, with the following fixed-effect covariates: treatment, geographical region, modified New York (mNY) classification, the maintenance period baseline value, visit as a fixed-effect factor, treatment group by visit interaction, and the maintenance period baseline value by visit interaction. MMRM analysis was conducted using the randomised set, comprising all randomised patients. The percentage of patients maintaining 50% improvement in BASDAI (BASDAI50) through the maintenance period was determined using a logistic regression model (with the following factors: treatment group, geographical region, and mNY classification) and nonresponder imputation. Statistical analyses were performed using SAS Version 9.3 (SAS Institute Inc., Cary, North Carolina, USA).

## RESULTS

### Baseline characteristics, sustained remission, and patient disposition

Baseline characteristics and patient disposition in the induction and maintenance periods and efficacy results from the induction period have been published [[13], [14], [15]].

Briefly, 736 patients with axSpA were enrolled to receive open-label CZP for 48 weeks in the induction period, with a mean (SD) age at study entry of 32.9 years (7.0) and symptom duration of 3.3 years (2.2). As previously reported, 323 patients (43.9%) achieved sustained remission at week 48 with open-label CZP 200 mg Q2W [13,14]. Ten of the 323 patients did not enter the maintenance period due to patient withdrawal or ineligibility. The remaining 313 patients were randomised in the maintenance period to continue with full-dose CZP (200 mg Q2W; n = 104) or receive reduced-dose CZP (200 mg Q4W; n = 105) or placebo (n = 104).

Patient disposition flow diagrams for the induction and maintenance periods, which include reasons for discontinuing treatment, have been published [13,14]. In brief, 89.5% of patients (n = 659/736) completed the open-label induction period. During the maintenance period, the percentages of patients who experienced a flare and subsequently received open-label CZP 200 mg Q2W in the escape arm were 6.7% (n = 7/104) with full-dose CZP, 14.3% (n = 15/105) with reduced-dose CZP, and 70.2% (n = 73/104) with placebo. The percentages of patients who completed the maintenance period in the assigned treatment groups were 85.6% (n = 89/104) with full-dose CZP, 80.0% (n = 84/105) with reduced-dose CZP), and 23.1% (n = 24/104) with placebo.

At baseline in the maintenance period (ie, randomisation at week 48), patient-reported disease characteristics in the current analyses (ie, BASDAI total, BASDAI morning stiffness, BASDAI fatigue, BASFI, PtGADA, nocturnal spinal pain, and ASQoL scores) were generally comparable in the full-dose CZP, reduced-dose CZP, and placebo groups (Table 1). Patient-reported disease characteristics at baseline of the induction period (ie, week 0) are also shown in Table 1, only for patients who entered the maintenance period. Comparisons of these baseline data in the induction and maintenance periods demonstrate that PRO improvements were observed with CZP therapy during the induction period (Table 1), as previously reported [[13], [14], [15]].

**Table 1:** Disease characteristics at baseline in the induction period (week 0) and in the maintenance period (week 48) for patients randomised in the maintenance period (by randomised group)^a^

	Placebo^a^ (n = 104)	CZP 200 mg Q2W (n = 104)	CZP 200 mg Q4W (n = 105)
Induction period, mean (SD)			
BASDAI total	6.3 (1.3)	6.5 (1.4)	6.7 (1.5)
BASDAI morning stiffness	6.7 (1.7)	7.0 (1.8)	6.9 (1.9)
BASDAI fatigue	6.7 (1.5)	7.0 (1.7)	7.0 (1.6)
BASFI	4.8 (1.9)	5.2 (1.8)	5.3 (2.1)
PtGADA	6.3 (2.0)	6.9 (2.0)	6.9 (1.8)
Nocturnal spinal pain	6.4 (2.2)	7.1 (2.1)	7.0 (2.3)
ASQoL	10.0 (4.4)	10.5(4.4)	11.2 (4.6)
Maintenance period, mean (SD)			
BASDAI total	0.5 (0.6)	0.4 (0.5)	0.4 (0.5)
BASDAI morning stiffness	0.4 (0.7)	0.4 (0.6)	0.3 (0.6)
BASDAI fatigue	0.8 (1.1)	0.8 (1.2)	0.7 (1.2)
BASFI	0.5 (0.8)	0.4 (0.5)	0.3 (0.5)
PtGADA	0.4 (0.7)	0.5 (0.7)	0.3 (0.5)
Nocturnal spinal pain	0.3 (0.6)	0.3 (0.6)	0.2 (0.4)
ASQoL	1.0 (2.0)	0.5 (1.3)	0.5 (1.4)

ASQoL, Ankylosing Spondylitis Quality of Life; BASDAI, Bath Ankylosing Spondylitis Disease Activity Index; BASFI, Bath Ankylosing Spondylitis Functional Index; CZP, certolizumab pegol; PtGADA, Patient Global Assessment of Disease Activity; Q2W, every 2 weeks; Q4W, every 4 weeks.

Additional disease characteristics and demographics have been published [14].

a In the induction period, all patients were assigned to receive CZP 200 mg every Q2W (after a loading dose of CZP 400 mg at weeks 0, 2, and 4) for 48 weeks. In the maintenance period, patients were either withdrawn from CZP therapy (receiving placebo from week 48) or received CZP 200 mg Q2W (full dose) or CZP 200 mg every 4 weeks (Q4W; reduced dose).

### Treatment compliance and concomitant medications throughout the maintenance period

In the maintenance period, the number of injections deviated from the scheduled number by a mean (SD) of −0.4 (0.8) for full-dose CZP, −0.3 (0.8) for reduced-dose CZP, and −0.2 (0.5) for placebo. Mean (SD) compliance ratios were 0.98 (0.04), 0.98 (0.6), and 0.98 (0.05), respectively.

Concomitant NSAIDs were used by 84.0% of patients in the maintenance period (81.7%, 87.6%, and 82.5% in the full-dose CZP, reduced-dose CZP, and placebo groups, respectively). As shown in Table 2 for patients who entered the maintenance period, ASAS-NSAID intake scores were higher at baseline in the induction period (week 0) than at the start of the maintenance period (week 48); for instance, mean (SD) scores were 68.8 (44.5) and 56.6 (46.9) at the respective timepoints in the full-dose CZP group. When calculated at 4-week intervals, ASAS-NSAID scores remained stable across the maintenance period in both CZP groups (Table 2).

**Table 2:** ASAS-NSAID score at baseline in the induction period (week 0), at baseline in the maintenance period (week 48), and change in score from week 0 values for 4-week intervals across the maintenance period (by randomised group)^a^

ASAS-NSAID scores for all randomised patients in the maintenance period, mean (SD)	Placebo^a^ (n = 104)	CZP 200 mg Q2W (n = 104)	CZP 200 mg Q4W (n = 105)
Week 0 (induction period baseline)	71.7 (41.7)	68.8 (44.5)	75.9 (40.1)
Week 48 (maintenance period baseline)	58.9 (46.3)	56.6 (46.9)	70.2 (44.6)
Week 48-52	59.2 (46.1)	57.7 (46.4)	70.2 (44.5)
Week 52-56	59.9 (46.0)	57.0 (44.4)	71.2 (43.3)
Week 56-60	58.9 (45.7)	58.1 (44.1)	71.6 (42.9)
Week 60-64	58.1 (46.1)	57.6 (44.2)	72.5 (42.9)
Week 64-68	59.1 (48.5)	57.8 (44.2)	72.9 (42.6)
Week 68-72	61.1 (47.6)	58.0 (44.2)	72.4 (44.0)
Week 72-76	57.4 (50.2)	57.0 (44.1)	72.7 (43.1)
Week 76-80	63.4 (50.3)	58.0 (44.0)	72.6 (43.9)
Week 80-84	64.2 (51.6)	57.5 (44.3)	73.6 (43.9)
Week 84-88	68.1 (50.9)	58.3 (44.0)	73.4 (43.7)
Week 88-92	69.5 (51.4)	58.2 (44.2)	73.4 (43.7)
Week 92-96	62.5 (47.3)	57.4 (45.3)	71.0 (43.2)

ASAS-NSAID scores for patients who completed the maintenance period, mean (SD)	Placebo^a^ (n = 24)	CZP 200 mg Q2W (n = 89)	CZP 200 mg Q4W (n = 84)

Week 0 (induction period baseline)	74.8 (47.4)	69.1 (42.3)	79.6 (39.0)
Week 48 (maintenance period baseline)	71.0 (49.8)	58.5 (44.4)	74.1 (44.0)
Week 48-52	71.0 (49.8)	58.6 (44.4)	74.1 (44.0)
Week 52-56	71.0 (49.8)	58.6 (44.4)	73.7 (43.2)
Week 56-60	71.0 (49.8)	58.6 (44.3)	73.6 (43.1)
Week 60-64	71.0 (49.8)	58.5 (44.4)	74.4 (42.6)
Week 64-68	71.2 (49.6)	58.5 (44.4)	74.4 (42.6)
Week 68-72	71.9 (48.6)	58.7 (44.6)	74.1 (44.0)
Week 72-76	71.0 (49.8)	58.5 (44.4)	73.6 (43.2)
Week 76-80	71.1 (49.9)	59.1 (44.0)	74.5 (43.6)
Week 80-84	71.0 (49.8)	58.6 (44.3)	74.5 (43.4)
Week 84-88	71.1 (49.8)	58.7 (44.1)	74.2 (43.2)
Week 88-92	72.2 (50.8)	58.8 (44.3)	74.2 (43.2)
Week 92-96	71.0 (49.8)	59.1 (44.7)	74.2 (43.2)

ASAS, Assessment of SpondyloArthritis International Society; CZP, certolizumab pegol; NSAID, nonsteroidal anti-inflammatory drug; Q2W, every 2 weeks; Q4W, every 4 weeks.

a In the induction period, all patients were assigned to receive CZP 200 mg every Q2W (after a loading dose of CZP 400 mg at weeks 0, 2, and 4) for 48 weeks. In the maintenance period, patients were either withdrawn from CZP therapy (receiving placebo from week 48) or received CZP 200 mg Q2W (full dose) or CZP 200 mg every 4 weeks (Q4W; reduced dose).

Concomitant DMARDs were used by 22.1% of patients in the maintenance period (20.2%, 22.9%, and 23.3% in the full-dose CZP, reduced-dose CZP, and placebo groups, respectively).

### PROs with full-dose and reduced-dose CZP throughout the maintenance period

**Figure 1:**
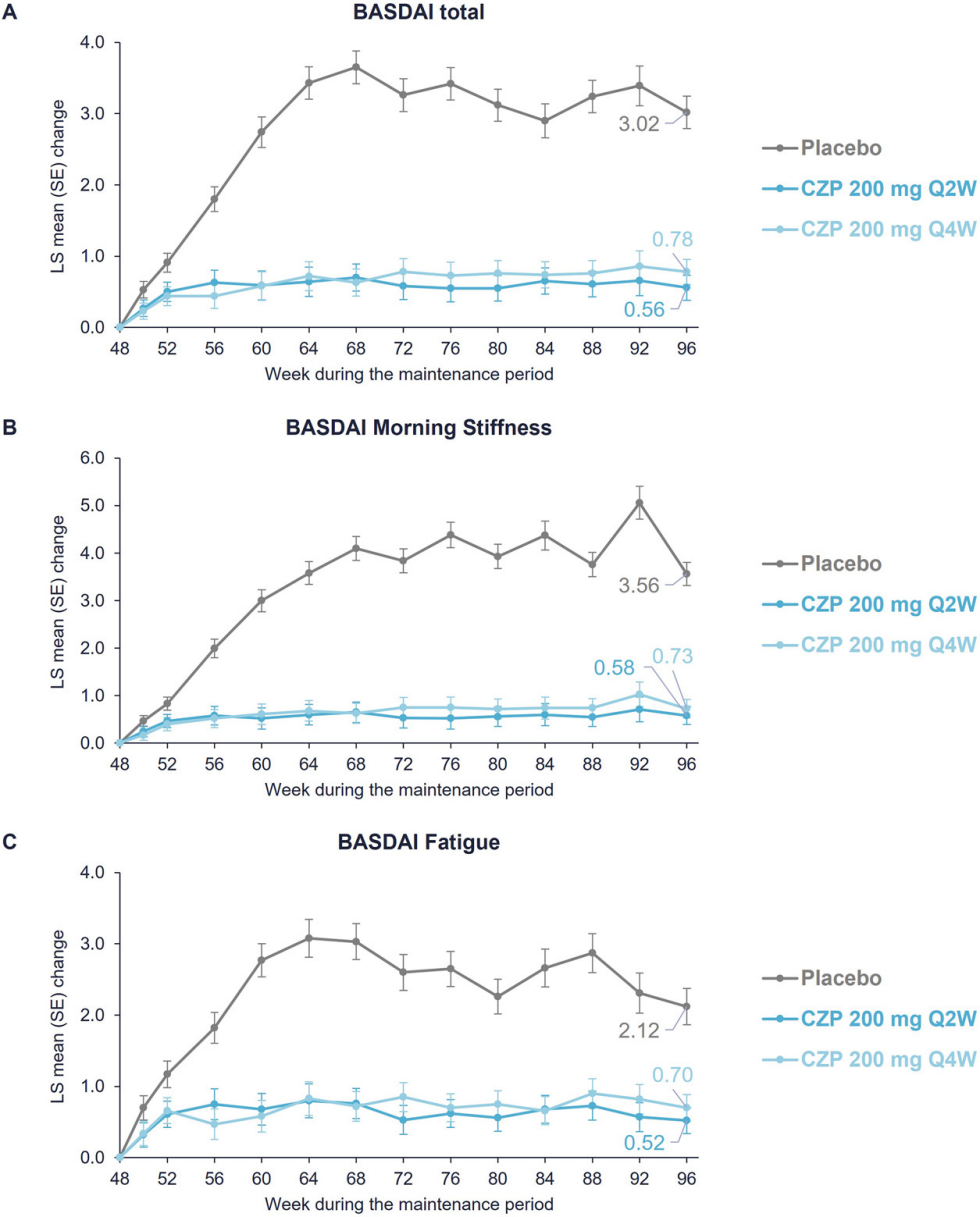
Change from week 48 to 96 in (A) BASDAI total, (B) BASDAI morning stiffness, and (C) BASDAI fatigue scores with full-dose CZP, reduced-dose CZP, and placebo. When analysed by MMRM, LS mean BASDAI total score changes were statistically significant at all time points from week 50 onwards for both CZP groups vs placebo (*P* < .001 from week 56). LS mean BASDAI morning stiffness score changes were statistically significant at week 50 with reduced-dose CZP (*P* < .05), and at all other time points for both CZP groups vs placebo (*P* < .001 from week 56). LS mean BASDAI fatigue score changes were statistically significant at week 50 with full-dose CZP (*P* < .05), and at all other time points for both CZP groups vs placebo (*P* < .001 from week 56). The numbers of patients randomised at week 48 to the full-dose CZP, reduced-dose CZP, and placebo groups were n= 104, n = 105, and n = 104, respectively. The numbers of patients who completed in the assigned treatment groups were n = 89, n = 84, and n = 24, respectively. BASDAI, Bath Ankylosing Spondylitis Disease Activity Index; CZP, certolizumab pegol; LS, least squares; MMRM, mixed model with repeated measures; Q2W, every 2 weeks; Q4W, every 4 weeks.

**Figure 2:**
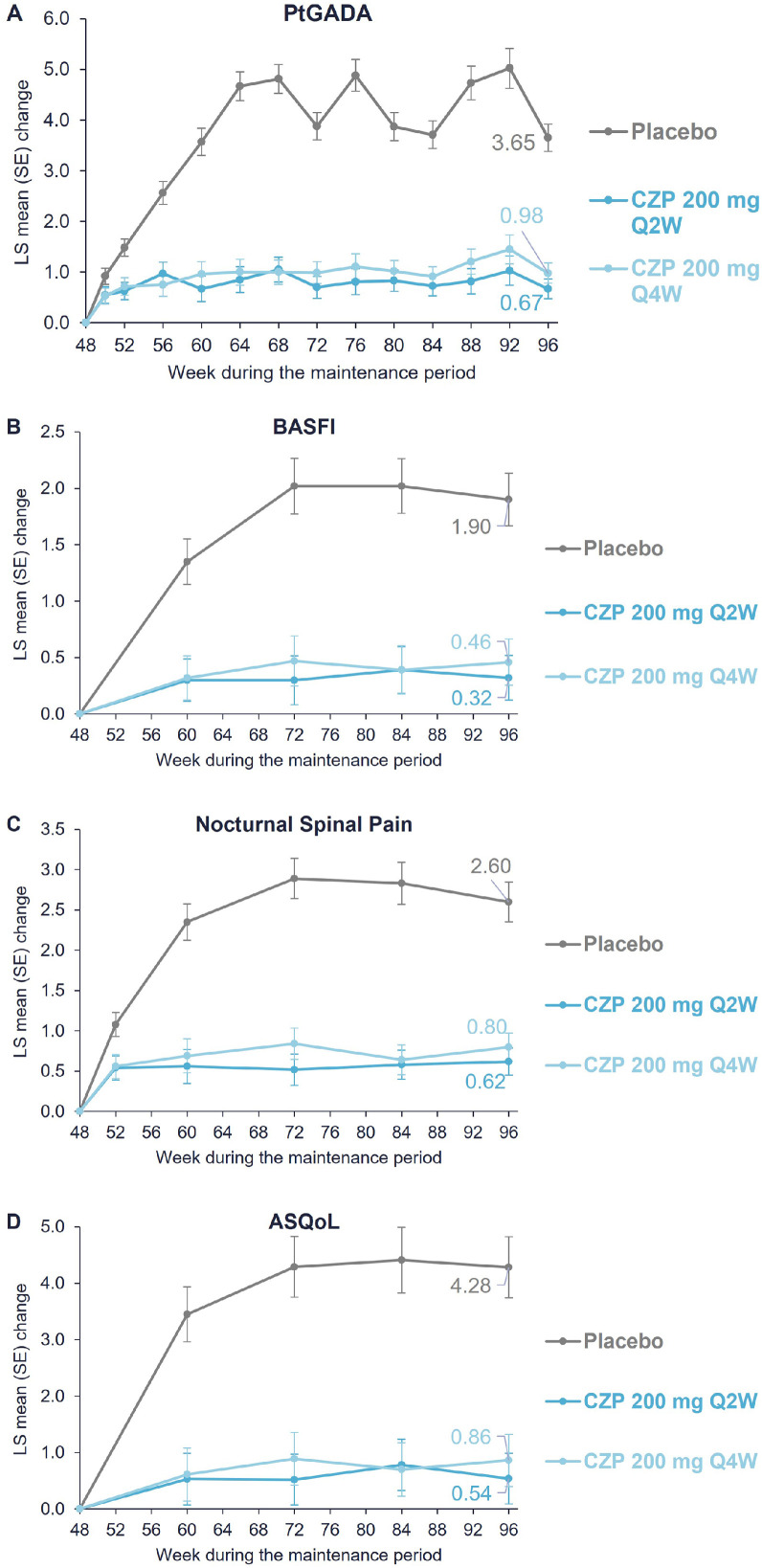
Change in score from week 48 to 96 in (A) PtGADA, (B) BASFI, (C) nocturnal spinal pain, and (D) ASQoL with full-dose CZP, reduced-dose CZP, and placebo. When analysed by MMRM, LS mean score changes were statistically significant at all time points for both CZP groups vs placebo (*P* < .001 from week 52 [PtGADA] and 60 [BASFI, nocturnal spinal pain, and ASQoL]). The numbers of patients randomised at week 48 to the full-dose CZP, reduced-dose CZP, and placebo groups were n = 104, n = 105, and n = 104, respectively. The numbers of patients who completed in the assigned treatment groups were n = 89, n = 84, and n = 24, respectively. ASQoL, Ankylosing Spondylitis Quality of Life; BASFI, Bath Ankylosing Spondylitis Functional Index; CZP, certolizumab pegol; LS, least squares; MMRM, mixed model with repeated measures; PtGADA, Patient Global Assessment of Disease Activity; Q2W, every 2 weeks; Q4W, every 4 weeks.

**Figure 3:**
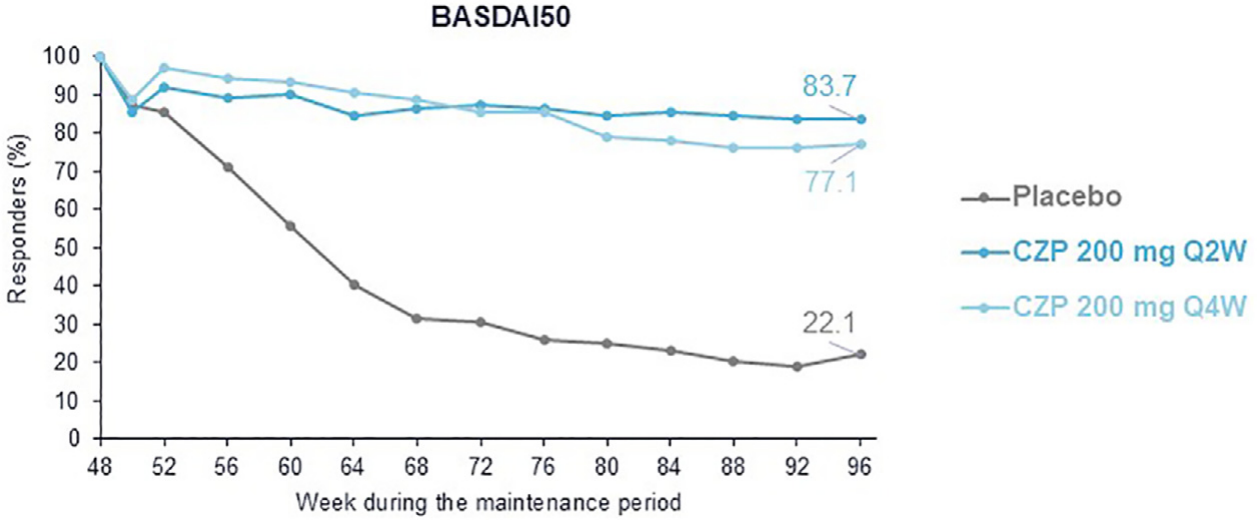
Change from week 48 to 96 in the percentage of patients with BASDAI50 maintained with full-dose CZP, reduced-dose CZP, and placebo. When analysed with logistic regression and nonresponder imputation, BASDAI50 changes were statistically significant at all time points from week 52 onwards for the full-dose CZP group vs placebo (*P* < .001 from week 56) and from week 56 onwards for the reduced-dose CZP group vs placebo (*P* < .001 from week 60). The numbers of patients randomised at week 48 to the full-dose CZP, reduced-dose CZP, and placebo groups were n = 104, n = 105, and n = 104, respectively. The numbers of patients who completed in the assigned treatment groups were n = 89, n = 84, and n = 24, respectively. BASDAI, Bath Ankylosing Spondylitis Disease Activity Index; CZP, certolizumab pegol; Q2W, every 2 weeks; Q4W, every 4 weeks.

All PROs demonstrated a similar pattern during the maintenance period, in prespecified assessments (BASDAI and BASFI) and *post hoc* assessments (PtGADA, nocturnal spinal pain, and ASQoL) (Figs 1-3, Supplementary Figs S1-S5, and Supplementary Table S3). For each PRO, in the CZP full-dose and reduced-dose groups, least squares (LSs) mean improvements were generally stable at each timepoint from week 48 to 96 (Figure 1, Figure 2). In both CZP groups, these stable PRO scores were statistically significantly different to PROs that worsened in the placebo group (ie, in patients withdrawn from CZP therapy at week 48 in the induction period). In the placebo group, LS mean scores of each PRO rapidly worsened from week 48, plateaued at approximately week 64 to 96, and were between 3-fold and 8-fold fold worse at week 96 than in the CZP full-dose and reduced-dose groups (Figure 1, Figure 2). From week 48 to 96, change in LS mean score with CZP full-dose and reduced-dose vs placebo (all *P* < .001) was, respectively: BASDAI total score 0.56 and 0.78 vs 3.02; BASDAI morning stiffness 0.58 and 0.73 vs 3.56; BASDAI fatigue 0.52 and 0.70 vs 2.12; BASFI 0.32 and 0.46 vs 1.90; PtGADA 0.67 and 0.98 vs 3.65; nocturnal spinal pain 0.62 and 0.80 vs 2.60; and ASQoL 0.54 and 0.86 vs 4.28.

Notably, although all PRO scores plateaued with placebo in the maintenance period (Figure 1, Figure 2), they did not return to the induction period baseline values (Table 1).

In responder analyses, the percentages of patients with BASDAI50 response maintained from week 48 through week 96 were statistically significantly greater in the CZP full-dose and reduced-dose groups (83.7% and 77.1%, respectively), compared with placebo (22.1%) (Fig 3).

## DISCUSSION

These prespecified and *post hoc* analyses from the maintenance period of C-OPTIMISE demonstrate that, in patients with axSpA in CZP-induced remission at week 48 in the induction period, reduced-dose CZP therapy maintained stable PRO score improvements through week 96, comparable to full-dose CZP. These findings were consistent across a range of PRO scales, reflecting maintained improvements in symptoms and functioning, including fatigue, morning stiffness, nocturnal spinal pain, and health-related QoL.

International guidelines recommend considering TNFi dose reduction for patients with axSpA in remission [8,9]. Our PRO findings across week 48 to 96, and published data showing maintenance of low disease activity with reduced-dose CZP during the same time period [14], support this recommendation [8,9]. Importantly, these guidelines also highlight the overarching principle of ‘best care’ based on ‘shared decision-making’ through informed dialogue between the patient and clinician [8,9]. This dialogue is critical to reach treatment decisions [8], particularly as there may be anxiety from the patient in terms of their QoL deteriorating with a reduced dose of CZP. Our findings should help facilitate such conversations, with reassurance that PROs are unlikely to be negatively impacted by the dose reduction of CZP. Our findings also support guideline recommendations to maximise long-term health-related QoL and the need to monitor PROs, as well as clinical findings, laboratory tests and imaging [8].

In the placebo group (ie, patients withdrawn from CZP therapy at week 48), worsening PRO scores plateaued from approximately week 64 and were maintained through week 96, although without returning to the average values observed at week 0 (baseline). Although this suggests persistence of some benefit of CZP therapy in the placebo group, another explanation may be that it is related to patient discontinuations. Importantly, our findings also demonstrate that continued treatment with either reduced-dose or full-dose CZP therapy is necessary for patients to experience the full benefits of CZP treatment.

The stable PRO score improvements across the maintenance period are consistent with previously reported findings, including maintenance of ASDAS between week 48 and 96 with reduced-dose and full-dose CZP therapy [14]. In addition to PROs, in the current analyses, we also demonstrated high rates of CZP treatment compliance and comparable use of concomitant medications (NSAIDs and DMARDs) per treatment group during the maintenance period. The percentages of patients treated with concomitant NSAIDs (84.0%) or DMARDs (22.6%) have been reported for the induction period (week 0-48) [13] and were comparable in the maintenance period (week 48-96). However, a reduction in average ASAS-NSAID score between week 0 and 48 reflects less usage (eg, lower dose or longer dosing intervals) with open-label CZP therapy during the induction period, which remained stable with double-blind CZP therapy throughout the maintenance period.

We do not report safety outcomes in the current paper. Previous analyses demonstrate that no new concerns were identified throughout the C-OPTIMISE study [13,14].

These analyses of the C-OPTIMISE study have notable strengths and limitations. A key strength is the consistency of improvements, maintained across multiple PRO scales that captured various aspects of the disease from the patients’ perspectives, contributing to the robustness of the findings. Similarly, the trajectory of PRO improvements across multiple timepoints in the maintenance period suggests that scores were stable with both CZP doses, although the possibility of fluctuations between timepoints cannot be discounted. It should also be noted that the current findings with reduced-dose CZP are applicable only to patients who achieved sustained remission during the open-label induction period, and thus may not be extrapolated to more refractory patients (ie, who did not achieve remission with initial treatment). One drawback is the limited number of patients who completed the maintenance period in the placebo group (23.1%), mainly due to the occurrence of flares that resulted in open-label treatment with full-dose CZP in the escape arm. However, as previously reported, improvements in disease activity and PROs (including BASDAI and BASFI) were observed after 12 weeks of escape treatment [14], and, in the current analyses across the maintenance period, statistically significant separation from placebo for PROs and high completion rates were observed with both CZP groups (85.6%, full-dose; 80.0% reduced-dose). Regarding the potential for further research, given the chronic nature of axSpA, longer-term data would be beneficial to assess outcomes during dose tapering, including from randomised controlled trials and real-world settings. Further research could also explore whether some patients are better suited to dose reduction than others and, if so, what predictors might help to inform personalised treatment strategies.

In conclusion, these findings, along with previously published data, demonstrate that reducing the CZP dose in patients with axSpA in sustained remission is likely a viable strategy to maintain remission or low disease activity without compromising PROs. These findings should offer reassurance to patients and physicians that patients’ QoL should not be impacted by a reduction in CZP dose. Further studies should evaluate whether CZP dose reduction in axSpA has an impact on other PROs, immunosuppression, and healthcare costs.

## Competing interests

AC has been a member of advisory boards or speaker's bureau for UCB, Novartis, Sanofi, AbbVie, Celgene, and Janssen. JW, LB, and MK are employees of UCB. TK, BH, and LB are employees and shareholders of UCB. KM received consultancy and speaker meetings for UCB, AbbVie, Novartis, Lilly, AZ, Janssen, and Roche. KG received grant/research support from NASS, Versus Arthritis, AbbVie, Pfizer, UCB, Novartis, Lilly, Celegene, Celltrion, Janssen, Gilead, and Biogen; Honoria/consultation fees from Novartis, AbbVie, UCB, Lilly, and Pfizer; Speaker’s bureau from Novartis, UCB, AbbVie, and Lilly; meeting expenses from AbbVie, Lilly, Roche, Novartis, and Pfizer, UCB; and is a shareholder of Rheumatology events.

## Appendix Supplementary materials

Image, application 1

## Footnotes

Supplementary material associated with this article can be found in the online version at doi:10.1016/j.ero.2026.100183.
