# Supplementary material for: Maintained improvement in patient-reported outcomes in early axial spondyloarthritis following certolizumab pegol dose reduction: findings from the randomised period of a phase 3b trial
Source: EULAR Rheumatol Open. 2026 May 7;2(2):100183. doi: 10.1016/j.ero.2026.100183 (PMC13425177; doi:10.1016/j.ero.2026.100183)

# **Online supplementary table and figures**

**Online supplementary table 1** Participating counties by region

| **Region** | **Country** |
| --- | --- |
| North America | United States of America |
| Western Europe | Belgium  France  Germany  The Netherlands  Spain  United Kingdom |
| Eastern Europe | Bulgaria  Czech Republic  Hungary  Poland  Romania |
| Asia | Taiwan  Turkey |

**Online supplementary table 2** Additional information on randomisation, study treatment and blinding

| **Randomisation** |
| --- |
| - Patients were enrolled during screening by the investigator or designee via an Interactive Voice or Web Response System, which was subsequently used to randomise patients at Week 48. - Randomisation was stratified by geographical region (**online supplementary table 1**). |
| **Provision of study treatment** |
| - CZP and placebo were supplied in 1 mL single-use glass prefilled syringes, with a 25G ½ inch thin wall needle. - Each syringe of CZP 200 mg/mL had an extractable volume of 1 mL in 10 mM sodium acetate and 125 mM sodium chloride. - Placebo was supplied in 0.9% saline. - Due to differences in presentation and viscosity, CZP and placebo were delivered in prefilled syringes, inside sealed boxes without information about the assigned treatment, to ensure blinding during the maintenance period. |
| **Study treatment administration and blinding** |
| - Suitable areas for subcutaneous administration were the lateral abdominal wall and upper outer thigh. - During the open-label induction period, after training at Weeks 2 and 4, patients self-administered CZP Q2W from Week 6 through Week 46 (except for on-site administration by site personnel at Weeks 12, 24, 32, and 36). - During the double-blind maintenance period, staff of the study sponsor, study site, and contract research organisation were blinded to treatment allocation, except for staff involved in supplies (e.g. coordinator, packager), staff monitoring pharmacy documentation, and site personal and home nurses who administered medication. - Study treatment was administered by unblinded home nurses during the maintenance period, except for on-site administration by unblinded site personnel at Weeks 48, 52, 60, 72, and 84. - In the event of an emergency, it was possible to determine the treatment and dose allocated. Appropriate persons (such as investigators) could obtain a password to access the IXRS information and thus unblind a patient’s treatment allocation. |

CZP, certolizumab pegol; IXRS, Interactive Voice or Web Response System; Q2W, every 2 weeks.

**Online supplementary table 3** Observed case analyses of median (range) change in patient-reported outcomes at Week 96 from Week 48 for patients randomised in the maintenance period (by randomised group)*

| **Change in patient-reported outcome, median (range)** | **Placebo***  **(n=24)** | **CZP 200 mg Q2W**  **(n=89)** | **CZP 200 mg Q4W**  **(n=84)** |
| --- | --- | --- | --- |
| BASDAI total | 0.8  (-0.8, 3.3) | 0.2  (-1.6, 2.8) | 0.0  (-2.0, 3.3) |
| BASDAI morning stiffness | 0.3  (-1.0, 4.5) | 0.0  (-1.5, 3.0) | 0.0  (-2.0, 4.5) |
| BASDAI fatigue | 1.0  (-1, 4) | 0.0  (-8, 4) | 0.0  (-5, 5) |
| BASFI | 0.2  (-0.7, 1.8) | 0.0  (-2.6, 2.5) | 0.0  (-0.8, 3.1) |
| PtGADA | 1.0  (-1, 4) | 0.0  (-4, 3) | 0.0  (-2, 6) |
| Nocturnal spinal pain | 0.5  (-3, 6) | 0.0  (-3, 4) | 0.0  (-1, 5) |
| ASQoL | 0.0  (-1, 9) | 0.0  (-5, 8) | 0.0  (-10, 6) |

*In the induction period, all patients were assigned to received CZP 200 mg every Q2W (after a loading dose of CZP 400 mg at Weeks 0, 2 and 4) for 48 weeks. In the maintenance period, patients were either withdrawn from CZP therapy (receiving placebo from Week 48) or received CZP 200 mg Q2W (full dose) or CZP 200 mg every four weeks (Q4W; reduced dose).

The numbers of patients randomised at Week 48 to the full-dose CZP, reduced-dose CZP, and placebo groups, were n=104, n=105, and n=104, respectively. The numbers of patients who completed in the assigned treatment groups were n=89, n=84, and n=24, respectively.

ASQoL, Ankylosing Spondylitis Quality of Life; BASDAI, Bath Ankylosing Spondylitis Disease Activity Index; BASFI, Bath Ankylosing Spondylitis Functional Index; CZP, certolizumab pegol; PtGADA, Patient Global Assessment of Disease Activity; Q2W, every 2 weeks; Q4W, every 4 weeks; SD, standard deviation.

**Online supplementary figure 1.** Change in BASDAI score at Week 96 from Week 48, with full-dose CZP, reduced-dose CZP, and placebo: (A) BASDAI total, (B) BASDAI Morning Stiffness, (C) BASDAI Fatigue.

The numbers of patients randomised at Week 48 to the full-dose CZP, reduced-dose CZP, and placebo groups, were n=104, n=105, and n=104, respectively. The numbers of patients who completed in the assigned treatment groups were n=89, n=84, and n=24, respectively.

BASDAI, Bath Ankylosing Spondylitis Disease Activity Index; CZP, certolizumab pegol; LS, least squares; MMRM, mixed model with repeated measures; Q2W, every 2 weeks; Q4W, every 4 weeks.


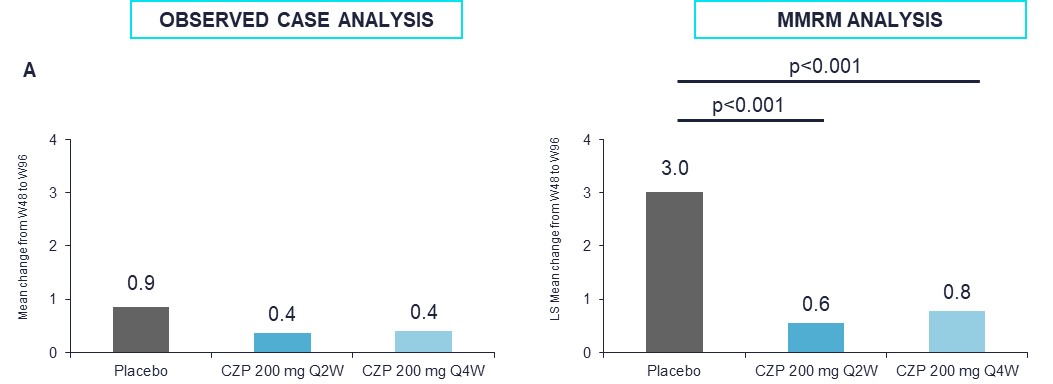


**
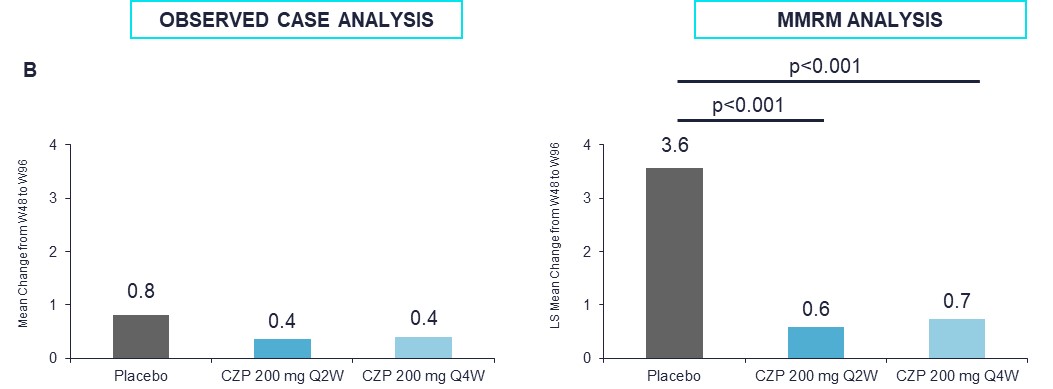
**

**
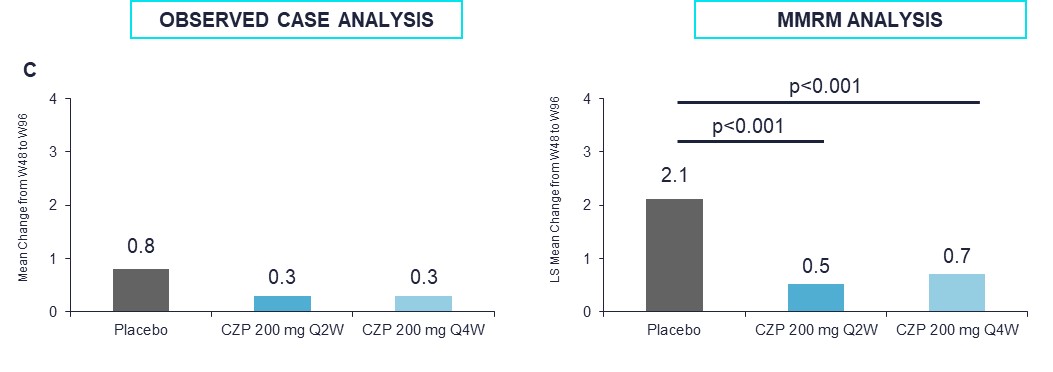
**

**Online supplementary figure 2.** Change in PtGADA score at Week 96, from Week 48, with full-dose CZP, reduced-dose CZP, and placebo.

The numbers of patients randomised at Week 48 to the full-dose CZP, reduced-dose CZP, and placebo groups, were n=104, n=105, and n=104, respectively. The numbers of patients who completed in the assigned treatment groups were n=89, n=84, and n=24, respectively.

CZP, certolizumab pegol; LS, least squares; MMRM, mixed model with repeated measures; PtGADA, Patient Global Assessment of Disease Activity; Q2W, every 2 weeks; Q4W, every 4 weeks.

**
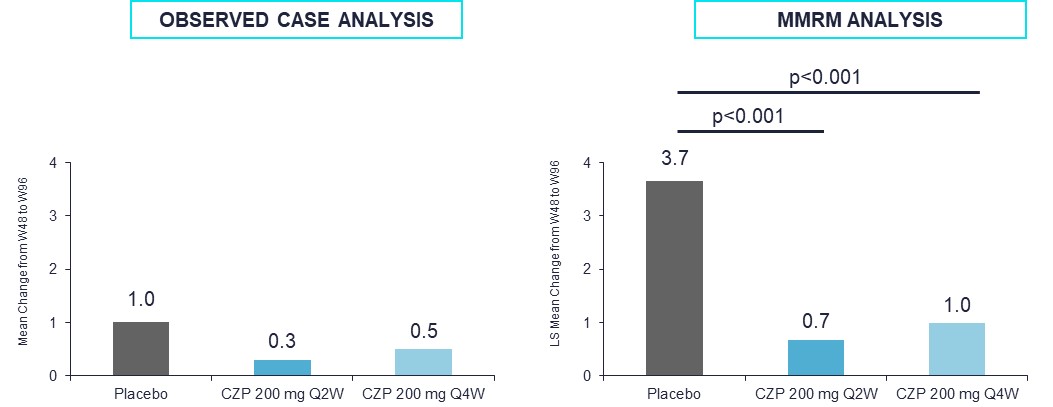
**

**Online supplementary figure 3.** Change in BASFI score at Week 96, from Week 48, with full-dose CZP, reduced-dose CZP, and placebo.

The numbers of patients randomised at Week 48 to the full-dose CZP, reduced-dose CZP, and placebo groups, were n=104, n=105, and n=104, respectively. The numbers of patients who completed in the assigned treatment groups were n=89, n=84, and n=24, respectively.

BASFI, Bath Ankylosing Spondylitis Functional Index; CZP, certolizumab pegol; LS, least squares; MMRM, mixed model with repeated measures; Q2W, every 2 weeks; Q4W, every 4 weeks.

**
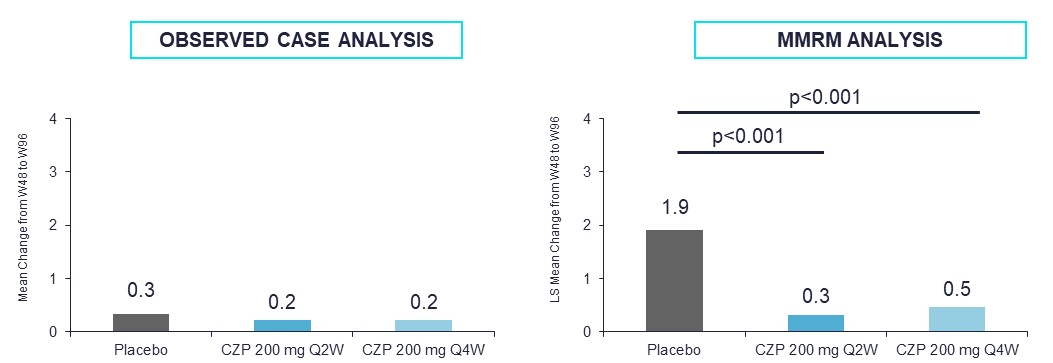
**

**Online supplementary figure 4.** Change in nocturnal spinal pain score at Week 96, from Week 48, with full-dose CZP, reduced-dose CZP, and placebo.

The numbers of patients randomised at Week 48 to the full-dose CZP, reduced-dose CZP, and placebo groups, were n=104, n=105, and n=104, respectively. The numbers of patients who completed in the assigned treatment groups were n=89, n=84, and n=24, respectively.

CZP, certolizumab pegol; LS, least squares; MMRM, mixed model with repeated measures; Q2W, every 2 weeks; Q4W, every 4 weeks.

**
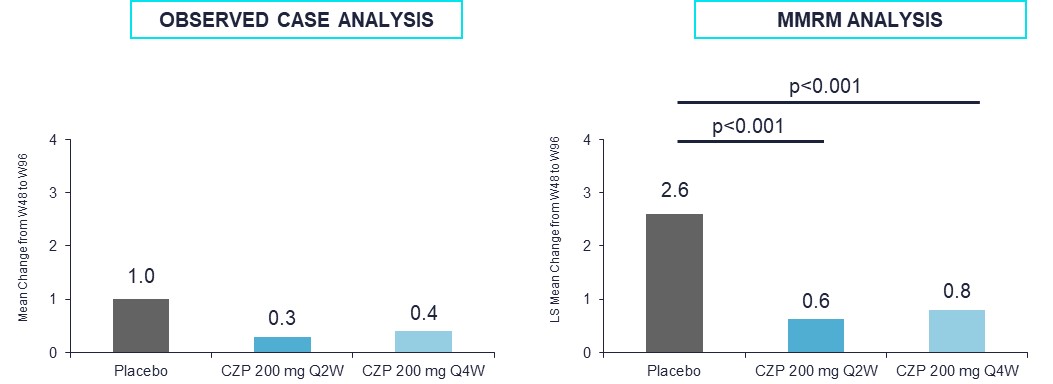
**

**Online supplementary figure 5.** Change in ASQoL score at Week 96, from Week 48, with full-dose CZP, reduced-dose CZP, and placebo.

The numbers of patients randomised at Week 48 to the full-dose CZP, reduced-dose CZP, and placebo groups, were n=104, n=105, and n=104, respectively. The numbers of patients who completed in the assigned treatment groups were n=89, n=84, and n=24, respectively.

ASQoL, Ankylosing Spondylitis Quality of Life; CZP, certolizumab pegol; LS, least squares; MMRM, mixed model with repeated measures; Q2W, every 2 weeks; Q4W, every 4 weeks.


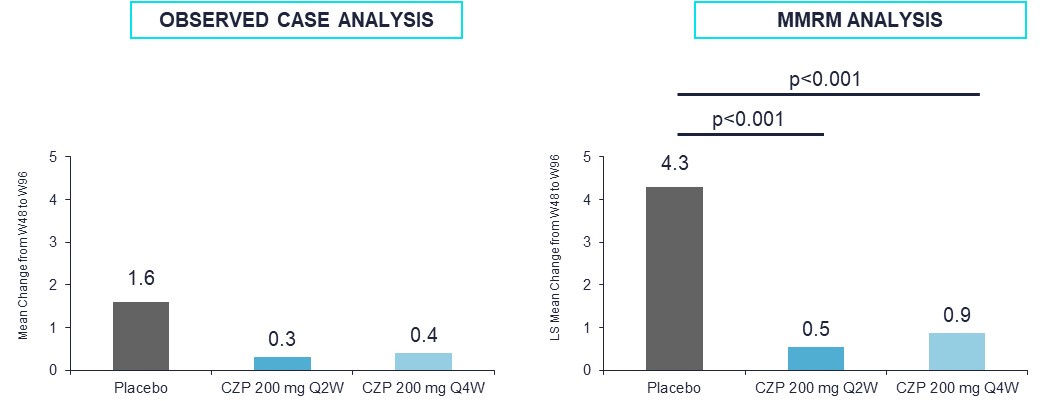

Supplement: Supplementary file 1 [file mmc1.docx]
